# Next-generation sequencing applications in food science: fundamentals and recent advances

**DOI:** 10.3389/fbioe.2025.1638957

**Published:** 2025-08-20

**Authors:** Joel Tigrero-Vaca, Byron Díaz, Ganyu Gu, Juan Manuel Cevallos-Cevallos

**Affiliations:** ^1^ Escuela Superior Politécnica del Litoral, ESPOL, Centro de Investigaciones Biotecnológicas del Ecuador (CIBE), Guayaquil, Ecuador; ^2^ Environmental Microbial and Food Safety Laboratory, USDA ARS, Beltsville, MD, United States

**Keywords:** next-generation sequencing, food microbiome, fermentation, food safety, food authentication

## Abstract

Next-generation sequencing (NGS) has revolutionized food science, offering unprecedented insights into microbial communities, food safety, fermentation, and product authenticity. NGS techniques, including metagenetics, metagenomics, and metatranscriptomics, enable culture-independent pathogen detection, antimicrobial resistance surveillance, and detailed microbial profiling, significantly improving food safety monitoring and outbreak prevention. In food fermentation, NGS has enhanced our understanding of microbial interactions, flavor formation, and metabolic pathways, contributing to optimized starter cultures and improved product quality. Furthermore, NGS has become a valuable tool in food authentication and traceability, ensuring product integrity and detecting fraud. Despite its advantages, challenges such as high sequencing costs, data interpretation complexity, and the need for standardized workflows remain. Future research focusing on optimizing real-time sequencing technologies, expanding multi-omics approaches, and addressing regulatory frameworks is suggested to fully harness NGS’s potential in ensuring food safety, quality, and innovation.

## 1 Introduction

In recent years, food manufacturing, processing, and distribution have evolved to enhance efficiency and meet global consumer demands. However, these advances bring new challenges, including contamination risks from complex supply chains, emerging foodborne pathogens, and increasing consumer expectations for safety, transparency, and sustainability. To address these issues, food science research must integrate modern microbiology tools, which provide insights into microbial ecology, metabolism, and genetics (Kumar et al., 2021).

Microorganisms, such as bacteria and fungi, play an important role in food production, preservation, safety and quality. Advancements in nucleic acid sequencing technologies have significantly improved food microbiology research, allowing for rapid and precise microbial identification, pathogen detection, and food authenticity assessment (Marchelli et al., 2012). Haga clic o pulse aquí para escribir texto. Next-generation sequencing (NGS) has expanded the ability to characterize microbiomes within complex food matrices through whole-genome sequencing (WGS), metagenetics, metagenomic and metatranscriptomic analysis (Loman and Pallen, 2015; Jagadeesan et al., 2019). Amplicon sequencing is a common approach of metagenetics to examine the diversity of microorganisms by amplification and sequencing of targeted genes or fragments (Carugati et al., 2015). Metagenomics involves untargeted genomic analysis of mixed microbial communities, while metatranscriptomics studies the collective transcriptomes of given habitats (Quince et al., 2017; Ojala et al., 2023). These sequencing approaches allow for deep taxonomic identification, strain-level genome reconstruction, and microbial community characterization (Ferrocino et al., 2023).

Since its introduction in the mid-2000s, NGS has transformed food microbiology research, reducing sequencing costs while improving throughput and accuracy (Goodwin et al., 2016; Heather and Chain, 2016). Continuous advancements in sequencing technologies and bioinformatics have made microbial genomics more accessible, allowing for deeper taxonomic identification and function analysis of food microbiomes (Cao et al., 2017; Cardinali et al., 2017; Van Dijk et al., 2018). This review aims to provide a comprehensive overview of NGS technologies and methodologies, highlighting their applications and potential in food science, and discussing how these innovations are shaping the future of this field.

## 2 NGS platforms used in food science

Microbial genome sequencing is now a standard tool in food microbiology research. This is largely owing to advances in NGS technologies, which have made sequencing faster, more accurate, and more affordable (Jagadeesan et al., 2019). Current widely used sequencing technologies can be placed in two major categories: short-read and long-read platforms. Short-read sequencing technologies, such as those developed by Illumina and Ion Torrent, typically rely on sequencing by synthesis (SBS) of complementary DNA strands. In Illumina sequencing, DNA fragments undergo clonal amplification via bridge PCR, followed by reversible terminator-based sequencing (Ambardar et al., 2016). Illumina systems range from benchtop sequencers like ISeq and MiSeq to production-scale sequencers like HiSeq and NovaSeq (Kulski, 2016). Similarly, Ion Torrent sequencing employs sequencing by synthesis approach, but detects nucleotide incorporation through changes in pH, as hydrogen ions are released during DNA polymerization. This approach eliminates the need for optical detection, using platforms such as the Ion PGM Dx and Ion GeneStudio S5 (Tripathi et al., 2019; Hu et al., 2021).

Long-read sequencing technologies offer longer read lengths (typically >10 kb) and real-time analysis, expanding the scope of microbial genome studies (Hu et al., 2021). Pacific Biosciences (PacBio) uses single-molecule real-time (SMRT) sequencing, allowing DNA replication without PCR amplification (Ardui et al., 2018). Oxford Nanopore sequencing measures electrical conductivity as nucleic acids pass through a nanopore, offering long-read sequences (Chen et al., 2023; Lamb et al., 2023).

The main characteristics and applications in food science of each type of sequencing platform are presented in Table 1.

**TABLE 1:** Main characteristics of NGS platforms and their applications in food science.

NGS technology	Principle	Advantages	Disadvantages	Food science applications	References for applications
Illumina	Sequencing by synthesis	High throughput and accuracy	Short reads, high initial investment (Petersen et al., 2019)	Metagenetics for evaluating food quality and safety	Anagnostopoulos et al. (2022), 2023; Díaz Cárdenas et al. (2024)
				Metagenomics for characterizing microbial dynamics of food fermentation processes	Pothakos et al. (2020), Kim et al. (2021), Lima et al. (2022)
				Metatranscriptomics for flavor formation during food fermentation	Xiao et al. (2021), Pan et al. (2022), Xiong et al. (2024)
				Metagenetics in food fermentation for starter culture development	Pregolini et al. (2021), Tigrero-Vaca et al. (2022), Vaccalluzzo et al. (2022)
				Whole Genome Sequencing (WGS) of foodborne pathogens	Bai et al. (2024), Habib et al. (2024), Yang et al. (2024)
				Metagenomics for studying microbial dynamics during meat processing	Barcenilla et al. (2024), Gaire et al. (2024), Sequino et al. (2024)
Ion Torrent	Sequencing by synthesis, detection of H^+^ ions	Small sample size needed. Sequencing takes 2–3 h	Short reads, relatively higher error rate (Shetty et al., 2019)	Metagenetics for seafood quality and safety assessment	Lira et al. (2020)
				Metagenetics for investigating the quality of dairy products	Syromyatnikov et al. (2020), Güley et al. (2023)
				Metagenetics in fermented foods for starter culture assessment	Ferrara et al. (2021)
PacBio	Single-molecule real-time (SMRT) sequencing	Long reads, high accuracy, minimal bias	High initial investment, large sequencer size (Petersen et al., 2019; Ling et al., 2023)	Metagenetics for analyzing the quality of dairy products	Ma et al. (2018), Yang et al. (2020), Liang et al. (2022)
Nanopore	Nanopore electrical signal sequencing	Long reads, portability, easy to use, low capital cost	Relatively high error rates, however Nanopore technology is undergoing constant advancements leading to reduced error rates and enhanced read accuracy (van der Reis et al., 2023)	Metagenomics for identification of foodborne pathogens and antimicrobial resistance genes (AMR)	Solcova et al. (2021), Lee et al. (2024)
				Metagenetics for spoilage microorganism detection in breweries	Kurniawan et al. (2021), Shinohara et al. (2021)
				Metagenetics for evaluating food quality during storage	Gu et al. (2024)
				Metagenomics to analyze microbial communities in fermented foods	Kharnaior and Tamang (2022), Tamang et al. (2022)
				WGS of foodborne pathogens	Martínez-Álvarez et al. (2024), Wang et al. (2024)

## 3 Process of NGS analysis in foods

**FIGURE 1:**
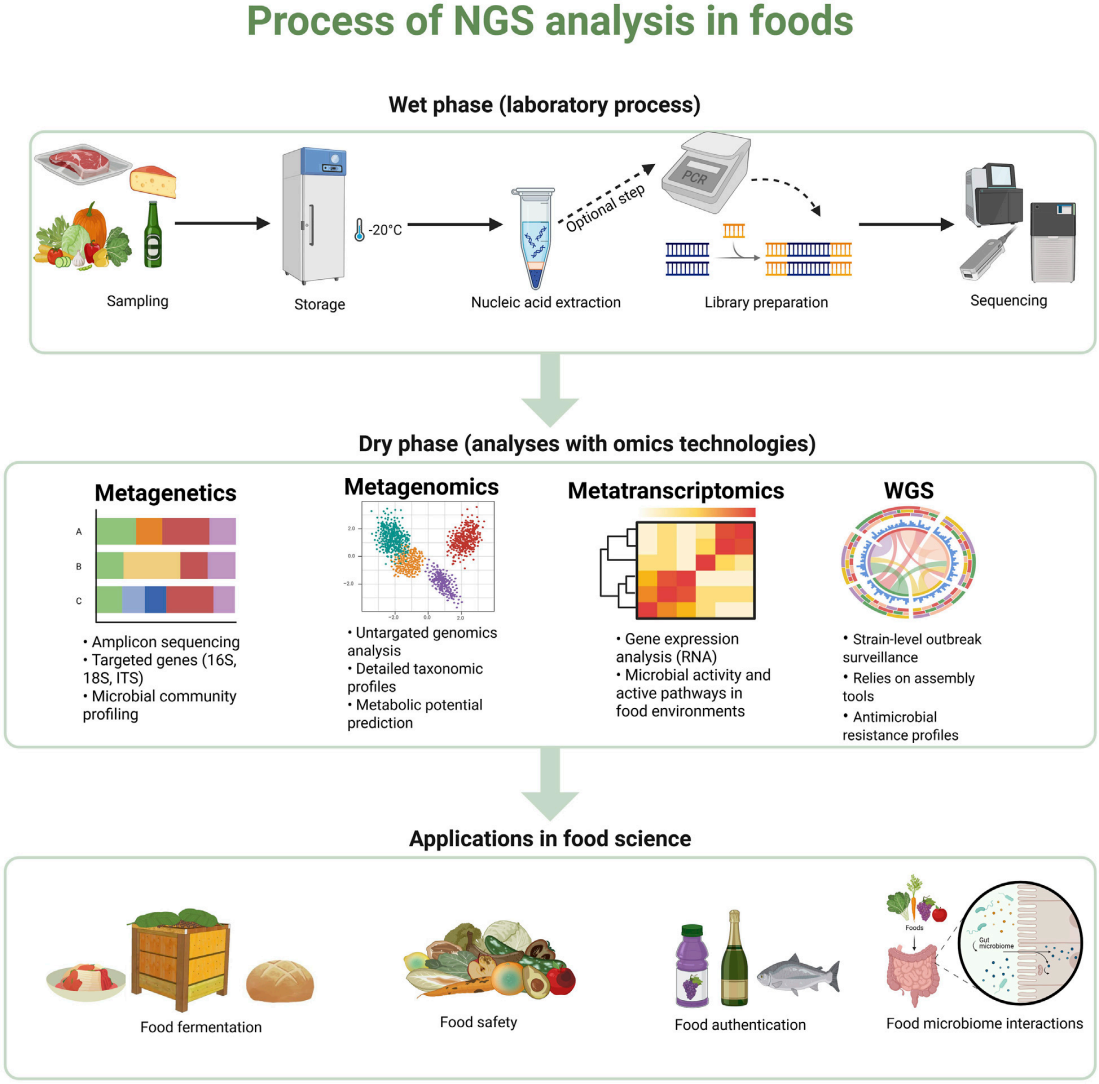
Schematic representation of the NGS workflow in food science, illustrating key steps including sample preparation and storage, DNA/RNA extraction, library preparation, sequencing, and the use of bioinformatics tools for various types of analysis including metagenetics, metagenomics, metatranscriptomics and WGS. Created using BioRender: https://BioRender.com.

Irrespective of sequencing technology and platform, each NGS operation includes two main phases: wet and dry. The wet phase refers to the laboratory process, which includes four key steps: (i) collecting and storing samples, (ii) extracting nucleic acids, (iii) library preparation including targeted gene and optional amplification, and (iv) library loading and sequencing run. The dry phase, on the other hand, focuses on the computational analysis of the sequencing data. A schematic overview of this process is illustrated in Figure 1.

### 3.1 Sample collection and storage

In general, similar considerations and precautions should be applied for food samples subjected to NGS and to conventional microbiological analyses to ensure sample representativeness and integrity. The quantity of samples and repetitions can significantly impact data accuracy and reproducibility. Hence, researchers need to balance the desire for large repetitions and the costs and feasibility of processing these samples. This is especially challenging for food sampling, as raw material microbiota can vary greatly among samples and can change during processing (Sadurski et al., 2024). Both probability (random) and non-probability (non-random)-based approaches have been proven effective in designing sampling schemes tailored to the research question and food type (Taherdoost, 2016).

For food samples to be analyzed using NGS, changes in cell cultivability is not a major concern, and therefore, harsher conditions can be applied, such as snap freezing, rapid drying, or even certain chemical preservatives can be applied to prevent continued microbial growth or other changes that alter the sample nucleic acid profiles (Fricker et al., 2019).

Proper storage is important to prevent nucleic acid degradation or microbial growth (Kazantseva et al., 2021). To mitigate these risks, food samples are usually cooled to 4 °C or frozen at −20 °C or −80 °C, depending on the available facilities (Lear et al., 2018). In contrast, shelf-stable products like freeze-dried cheonggukjang, a fermented soybean food of Korea, can be stored at room temperature in moisture-free conditions, due to their inherent microbial and chemical stability (Tamang et al., 2022).

Sampling must also account for different processing stages, which influence microbial composition. For instance, Barcenilla et al. (2024) used pooled swabs to improve microbial recovery from raw meat surfaces, while tailoring sampling techniques such as direct sampling or surface swabbing for various end products like fermented sausages or cured meats. Similarly, studies like Gaire et al. (2024) and Sequino et al. (2024) have emphasized the importance of stage-specific sampling to capture shifts in microbial diversity and antimicrobial resistance.

Ultimately, successful NGS-based food science studies rely on thoughtful sampling design and proper storage. Researchers should tailor these steps to the specific food matrix, processing stage, and study goals to ensure meaningful and reproducible results.

### 3.2 Extraction of nucleic acids

Nucleic acid extraction from food matrices is a vital process in NGS of food products enabling the detection and study of genetic material (Tan and Yiap, 2009). Nucleic acid extraction methods consist of three steps: lysis, purification, and nucleic acid recovery. Furthermore, extraction can be performed using conventional protocols or commercially available kits, with the choice of method depending on sample complexity and study objectives (Sajali et al., 2018).

Cell lysis is carried out to break open microbial cells and release nucleic acids. Lysis methods can be chemical, enzymatic, mechanical, or a combination, depending on the complexity of the matrix (Lever et al., 2015). For instance, enzymatic lysis combined with mechanical disruption has been effectively applied to romaine lettuce (Gu et al., 2024) and coffee (Pothakos et al., 2020) illustrating the importance of method customization based on the nature of the sample.

After cell lysis, nucleic acid purification is performed to separate DNA or RNA from cellular debris, proteins, and inhibitors, typically using liquid-liquid (LLE) or solid-phase extraction methods (SPE) (Ruggieri et al., 2016). For example, the CTAB and chloroform LLE method was used by Lima et al. (2022) to isolate DNA from fermented cacao and by (Xiao et al., 2022) for RNA isolation from Sichuan paocai, highlighting the continued relevance of traditional reagents in specific contexts. SPE kits utilizing silica-based filters reduce reliance on organic solvents and enhance efficiency in nucleic acid recovery (Emaus et al., 2020). These kits have been applied to extract DNA from raw meat swabs (Barcenilla et al., 2024; Gaire et al., 2024) and fermented soybean products (Kharnaior and Tamang, 2022; Tamang et al., 2022) showing their versatility. However, effectiveness can vary depending on sample composition, emphasizing the need for further optimization, particularly for high-fat or polyphenol-rich matrices.

Following lysis and purification, nucleic acid recovery is carried out by pelleting the nucleic acids through centrifugation, followed by resuspension in a suitable buffer. Nuclease-free water or 1× TE buffer is commonly used, with TE offering added protection (Schenk et al., 2023).

In conclusion, nucleic acid extraction is crucial for NGS-based studies on food science, employing diverse methods to ensure DNA/RNA quality and quantity. Tailoring techniques to specific samples enhances analytical reliability and accuracy, reinforcing confidence in research findings. Table 2 provides an overview of nucleic acid extraction techniques utilized in food science.

**TABLE 2:** Nucleic acid extraction methods utilized in food science.

Extraction method	Description	Advantages	Challenges	Food matrices	Example kits/References
CTAB and chloroform-based extraction	A variation of the phenol-chloroform method using CTAB for plant material	Effective for isolating DNA from challenging matrices	Use of hazardous chemicals	Fermented foods	Xiao et al. (2021), Lima et al. (2022)
Enzymatic lysis + mechanical disruption	Enzymatic lysis combined with mechanical methods to break down tough food matrices	Efficient for complex samples	Time-consuming; may require optimization	Leafy vegetables, fermented foods	Pothakos et al. (2020), Gu et al. (2024)
Silica-based spin columns-DNA kits	Commercial kits using silica columns to purify DNA from food samples	Safe, automated, and reproducible; fast process	More expensive than other methods	General use for various food matrices	NucleoSpin Food DNA kit Macherey-Nagel); (Kharnaior and Tamang, 2022; Tamang et al., 2022), DNeasy PowerSoil Pro kit (Qiagen) (Barcenilla et al., 2024; Gaire et al., 2024)
Silica-based spin columns-RNA kits	Commercial kits using silica columns to isolate RNA	RNA protection; high yield; compatible with downstream applications	Requires strict RNase-free conditions	Fermented foods, fruits, dairy products	Maxwell 16 LEV simply RNA tissue kit (Promega) (Jo et al., 2021), RNeasy Plant Mini Kit (Qiagen) (Quijada et al., 2022), InnuSPEED Bacteria/Fungi RNA kit (Analytik Jena) (Quijada et al., 2022)

### 3.3 Targeted gene and optional amplification

NGS in food science often incorporates a PCR amplification step to selectively enrich target marker genes from complex mixtures of genomic DNA. For instance, 16S ribosomal RNA (rRNA) gene sequencing is a targeted approach where PCR is used to amplify specific hypervariable regions of the 16S rRNA gene from diverse bacterial populations present in food samples (Abellan-Schneyder et al., 2021). This amplification enables in-depth profiling of microbial communities, which is essential for assessing food safety, quality, and shelf-life.

In addition to the 16S rRNA gene, other marker genes such as the internal transcribed spacer (ITS) region for fungal identification and the 18S rRNA gene for eukaryotic microorganisms are also routinely targeted for amplicon sequencing (Banos et al., 2018). By selectively amplifying these marker genes, researchers can generate high-quality, focused libraries for NGS, ultimately providing deeper insights into the microbial ecology of food products and aiding in effective monitoring of potential contaminants (Fagerlund et al., 2021).

Additionally, marker genes play a crucial role in food authenticity determination by enabling the detection of species-specific DNA sequences to verify the origin and composition of food products (Vishnuraj et al., 2023). Commonly used markers include mitochondrial genes such as cytochrome b (*cyt b*) and cytochrome c oxidase I (COI) for identifying animal species, as well as nuclear genes like the *rbcL* and *matK* genes for plant species authentication (Grazina et al., 2020; Afifa et al., 2021; Zhao Z. et al., 2024). The cyt b and COI genes encode proteins involved in mitochondrial respiration and are favored for their high interspecies variability (Antil et al., 2023; Tutuş et al., 2025). In plants, rbcL encodes the large subunit ribulose-1,5-bisphosphate carboxylase/oxygenase (RuBisCO) essential for photosynthesis, while matK encodes maturase K, involved in chloroplast RNA splicing and known for its high substitution rate and discriminatory power at the species level (Jia et al., 2025). These genetic markers help prevent food fraud, ensure label accuracy, and protect consumers from misrepresentation or adulteration of food products.

In addition to targeted gene amplification, some NGS applications in food science, such as shotgun metagenomics, incorporate optional PCR amplification during library preparation to enhance sequencing efficiency. For example, the Nextera XT DNA Library Preparation Kit employs enzymatic fragmentation followed by PCR amplification to generate sequencing-ready libraries, particularly when working with food samples containing low-abundance of target DNA (Kim et al., 2013). This approach facilitates comprehensive microbial profiling and functional gene analysis in food samples. However, as seen in metagenomic studies like the genetic characterization of dengue viruses (Lizarazo et al., 2019), excessive PCR cycles could introduce additional biases and duplicates, potentially affecting sequencing accuracy.

### 3.4 Library preparation

NGS library preparation involves processing nucleic acids (RNA or DNA) to align with the chosen sequencing platform (Head et al., 2014). This includes fragmentation, multiplexing, normalization and adapter ligation (Abdi et al., 2024). While amplicon sequencing relies on short DNA fragments of target regions selectively amplified via PCR, shotgun sequencing requires fragmentation of total DNA using sonication, enzymatic digestion, or mechanical shearing (Srinivas et al., 2022). On the other hand, for RNA sequencing in metatranscriptomic analyses, mRNA molecules are converted to cDNA fragment by reverse transcription, using either poly(A) tailing (targeting eukaryotes) or random priming (targeting prokaryotes) (Saliba et al., 2014). Short-read platforms like Illumina favor fragments under 450 bp, whereas long-read platforms such as PacBio and Oxford Nanopore Technologies (ONT) require high molecular weight (HMW) DNA, sometimes sheared to ∼20 kb for improved sequencing efficiency (Madoui et al., 2015; Ribarska et al., 2022).

Multiplexing, or indexing, allows multiple libraries to be pooled and sequenced together using unique index sequences. To ensure equal representation, libraries must first be normalized to the same DNA/RNA concentration before being pooled in equal volumes (Muller et al., 2019). However, challenges like index hopping (where reads are misassigned) can introduce errors, particularly in Illumina platforms with patterned flow cells. Unique dual indexing and nested metabarcoding help mitigate these issues (MacConaill et al., 2018; Guenay-Greunke et al., 2021).

Adapter ligation attaches platform-specific sequences to DNA fragments, enabling binding to flow cells. Illumina uses adapters for anchoring, whereas PacBio employs hairpin adapters for circular DNA sequencing. ONT ligates adapters that guide DNA strands through nanopores (Oikonomopoulos et al., 2020; MacKenzie and Argyropoulos, 2023). Amplicon sequencing on Illumina integrates adapters directly during PCR, bypassing the ligation step (Glenn et al., 2019).

Minimizing contamination is critical, as microbial DNA from reagents can bias NGS analyses. Therefore, proper laboratory practices, negative controls, and mock microbial communities help reduce this risk (Salter et al., 2014). Advances in automation and quality control continue to enhance the reliability and efficiency of NGS library preparation in food science applications.

### 3.5 Sequencing

NGS methods for food microbiome analysis can be broadly divided into targeted (amplicon-based) and random (shotgun) sequencing approaches, each serving different analytical goals (Sekse et al., 2017).

Amplicon sequencing, also known as targeted sequencing, focuses on specific genetic markers, such as the 16S rRNA gene for bacteria, ITS regions for fungi, and COI genes for metazoans (Francioli et al., 2021). This approach, using platforms like Illumina MiSeq and Ion Torrent PGM is widely adopted due to cost-effectiveness and established analytical pipelines (Lira et al., 2020; Syromyatnikov et al., 2020; Anagnostopoulos et al., 2022; 2023; Güley et al., 2023). However, PCR-induced biases in primer selection can significantly affect microbiota characterization (Sergeant et al., 2012). In fungi, ITS length variation can distort community structure by favoring shorter fragments (De Filippis et al., 2017). Amplicon sequencing also lacks strain-level resolution, limiting food safety assessments (La Reau et al., 2023). To mitigate biases, researchers can consider alternative fungal-specific targets, such as 26S and 18S rRNA genes, for improved accuracy (Banos et al., 2018; Mota-Gutierrez et al., 2019).

Shotgun metagenomic sequencing, in contrast, sequences all DNA present in a sample, providing a comprehensive view of the genetic content, including functional genes, metabolic pathways, and antimicrobial resistance determinants (Tyagi et al., 2019). This approach enables deep characterization of food microbiomes, making it ideal for studying fermentation and resistome profiling (Pothakos et al., 2020; Kim et al., 2021; Kharnaior and Tamang, 2022; Lima et al., 2022; Tamang et al., 2022; Lee et al., 2024). However, it cannot easily distinguish live from dead cells. When viability is critical, combining metagenomics with culture-dependent techniques ensures accurate risk assessment (Lindner et al., 2024). Shotgun metagenomics requires robust computational resources for assembly and annotation (Tremblay et al., 2022). Platforms like Illumina NovaSeq and PacBio Sequel II offer high-throughput sequencing, while long-read technologies such as ONT and PacBio HiFi enhance genome assembly and resolution in complex food matrices (Zhang et al., 2022).

In addition, whole genome sequencing (WGS) and RNA sequencing (RNA-Seq) are also important for microbial analysis and food safety. Platforms used for WGS of foodborne pathogens include Oxford Nanopore MinION, for *Escherichia coli* characterization from meat products (Wang et al., 2024; Martínez-Álvarez et al., 2025). Illumina systems like NextSeq, HiSeq and NovaSeq, are widely employed for RNA-Seq for elucidating important microbial functions during food fermentation processes, as well as in transcriptomic studies of foodborne pathogens in fresh produce and related matrices (Xiao et al., 2021; Redding et al., 2024; Xiong et al., 2024; 2024; Ding et al., 2025). Comprehensive metatranscriptomic analyses of mRNA dynamics can also provide insights on microbial interactions in complex microbial communities or biological processes.

To enhance sequencing efficiency and accuracy, several strategies can be employed. Optimizing sample preparation helps minimize biases, especially in complex food matrices. Choosing the right sequencing platform ensures a balance between cost, read length, and throughput. Combining amplicon and shotgun approaches can provide complementary insights, and advancements in bioinformatics tools as discussed in the following section, such as machine learning-based classification and improved genome binning, can facilitate improved data interpretation for better tracking of foodborne pathogens and microbial ecology shifts.

### 3.6 Bioinformatic analysis

The vast amount of data generated by NGS requires robust bioinformatics pipelines for accurate analysis and interpretation. The bioinformatics workflow varies depending on the sequencing approach as well as the specific NGS platform used.

#### 3.6.1 Metagenetic analysis

Metagenetic analysis, including amplicon high-throughput sequencing, typically follows a standardized workflow, beginning with quality control (QC) and filtering to remove low-quality reads and sequencing artifacts. This step is commonly performed using tools like FastQC (Andrews, 2010) and DADA2 (Callahan et al., 2015) or USEARCH (Zhou et al., 2024), which help denoise sequences and cluster sequence readings into operational taxonomic units (OTUs) or amplicon sequence variants (ASVs). Taxonomic classification is then conducted using reference databases such as SILVA, Greengenes, or UNITE (for fungal ITS sequences) (Santamaria et al., 2012).

Common bioinformatics platforms, including QIIME2 (Bolyen et al., 2019) and Mothur (Schloss et al., 2009), streamline these processes and are widely applied in food microbiome studies. For instance, a study analyzing the bacterial diversity in fermented beverages used QIIME2 to assess microbial shifts during different stages of fermentation (Zhao X. et al., 2024). Similarly, UNITE was employed in a study to identify fungal communities in stored rice grains and their potential mycotoxin production (Qi et al., 2022).

The choice of reference databases and bioinformatics tools significantly impacts taxonomic classification accuracy, influencing the results of amplicon-based studies (De Filippis et al., 2017). While bacterial databases such as SILVA and Greengenes are well-curated, fungal databases remain comparatively less refined (Tedersoo et al., 2011). Enhancing genomic databases with foodborne microbial genomes and developing food-specific gene catalogs would improve taxonomic resolution and strengthen food microbiome research.

#### 3.6.2 Metagenomic analysis

Metagenomic analysis involves a more complex computational pipeline due to the high volume of sequencing data and the need for assembly. Quality control is performed using Trimmomatic (Bolger et al., 2014) or FASTP (Chen, 2023) to remove adapter sequences and low-quality reads. Reads are then assembled using tools like MEGAHIT (Li et al., 2015) or SPAdes (Bankevich et al., 2012), which reconstruct longer contigs from fragmented sequences.

Taxonomic and functional annotation is performed using databases such as Kraken2, MetaPhlAn, and KEGG, allowing researchers to identify microbial taxons, functional genes, and antimicrobial resistance markers in food samples (Nam et al., 2023). A study investigating the microbiome of food and environmental microbiomes in various meat processing facilities used Kraken2 for taxonomic classification, revealing that microbial communities change throughout processing, from raw materials to final products, with food contact surfaces significantly influencing the final microbiome (Barcenilla et al., 2024). Similarly, a metagenomic analysis of fermented vegetables used KEGG to map functional genes related to amino acid metabolism and probiotic activity (Yasir et al., 2022).

Long-read sequencing platforms, such as PacBio and Oxford Nanopore Technologies (ONT), improve metagenomic studies by generating high-continuity assemblies, enabling strain-level identification and plasmid detection in foodborne pathogens (Kwon et al., 2020; Espinosa et al., 2024). Tools like Flye (Kolmogorov et al., 2019) and Canu (Koren et al., 2017) are commonly used for long-read assembly, while Medaka and Racon aid in sequence polishing to improve accuracy (Lee et al., 2021). However, metagenomic data is inherently compositional, meaning relative abundances represent proportions rather than absolute feature loads (Morton et al., 2024). This perspective is crucial when interpreting pathogen detection, as any read set represents only a subset of the total community DNA. Proper contextualization ensures accurate conclusions about microbial presence and potential risks (Lindner et al., 2024).

#### 3.6.3 Metagenomic binning and downstream analysis of MAGs

Metagenome-assembled genomes (MAGs) provide insights into microbial community structure and function in food and in food production environments (Zhou et al., 2022). Metagenomic binning tools such as MetaBAT2 (Kang et al., 2019), CONCOCT (Alneberg et al., 2014), and MaxBin (Wu et al., 2014) group contigs into individual genome bins. A study examining microbial communities in artisanal cheese production successfully recovered high-quality MAGs using MetaBAT2, identifying bacterial strains responsible for flavor development (Walsh et al., 2020).

Quality assessment of MAGs can be performed using CheckM (Parks et al., 2015) and GUNC (Orakov et al., 2021) to ensure completeness and reduce contamination. Further downstream analysis includes genome annotation using tools like Prokka (Seemann, 2014) and EggNOG-mapper (Huerta-Cepas et al., 2017), which provide insights into microbial metabolism in food matrices.

#### 3.6.4 Functional profiling

Functional profiling is a key step in understanding microbial metabolism in food matrices. Tools like HUMAnN3, MEGAN, FAPROTAX, and PICRUSt2 predict metabolic functions based on sequencing data (Beier et al., 2017; Nam et al., 2023). For example, a study on fermented dairy products used HUMAnN3 to profile the microbial pathways involved in amino acid metabolism, linking them to health benefits (Yang et al., 2025).

#### 3.6.5 Metatranscriptomic analysis

Metatranscriptomics provides insights into microbial activity in food environments by analyzing RNA sequences. Bioinformatics tools streamline this process through key steps: quality control, assembly, annotation, and functional analysis. Preprocessing tools like FastQC, Trimmomatic, and SortMeRNA (Kopylova et al., 2012) ensure high-quality RNA-seq data. Read alignment and assembly rely on HISAT2 (Wen, 2017), STAR (Dobin et al., 2013), MEGAHIT, and Trinity (Haas et al., 2013),while functional annotation is performed using DIAMOND and Kaiju (Buchfink et al., 2015; Menzel et al., 2016). Differential expression and pathway analysis involve DESeq2, edgeR, and KEGG Mapper (Robinson et al., 2010; Anders and Huber, 2010; Kanehisa and Sato, 2020).

#### 3.6.6 WGS and antimicrobial resistance (AMR) analysis

WGS analysis, essential for tracing foodborne outbreaks, relies on assembly tools such as Unicycler (Wick et al., 2017) and annotation tools like Bakta (Schwengers et al., 2021) and ROARY (Page et al., 2015) for comparative genomics. For example, a WGS-based study on antibiotic-resistant *E*. *coli* from market chickens in Lima, Peru, used ROARY for comparative genomics to assess resistance gene distribution and potential transmission sources (Murray et al., 2021).

AMR surveillance in foodborne pathogens is critical for food safety. Databases like CARD (Comprehensive Antibiotic Resistance Database) (Alcock et al., 2023) and ResFinder (Florensa et al., 2022) help identify AMR genes, while tools such as AMRFinderPlus (Feldgarden et al., 2021) and ARIBA (Hunt et al., 2017) predict resistance profiles based on WGS and metagenomic data. A recent study utilizing nanopore sequencing-based metagenomics leveraged CARD to comprehensively identify AMR genes in food products, highlighting the prevalence of resistance determinants across diverse microbial communities (Lee et al., 2024). Similarly, ARIBA was used to analyze *E. coli* from store-bought produce, identifying diverse AMR genes and shedding light on potential resistance transmission through fresh vegetables (Reid et al., 2020).

There is an urgent need for standardized, open-access AMR databases to improve method comparison and surveillance. Advancing these technologies in microbiology labs will enhance detection, support personalized medicine, and streamline AMR monitoring.

#### 3.6.7 Advancing bioinformatics in food science

To enhance bioinformatics analysis, integrating multi-omics approaches (e.g., metagenomics with metabolomics or transcriptomics) can provide a deeper understanding of microbial activity and food quality (Balkir et al., 2021). Cloud-based bioinformatics platforms, such as MG-RAST (Keegan et al., 2016) and Galaxy (Goecks et al., 2010), enable scalable and user-friendly data analysis. The development of machine learning algorithms for microbial classification, AMR prediction, and genome annotation is further enhancing NGS applications in food safety and quality control. Table 3 provides an overview of the key bioinformatics tools and databases employed at various stages of NGS analysis in food science.

**TABLE 3:** Bioinformatics tools and workflows for NGS analysis in food science.

Analysis type	Tools/Databases	Workflow steps	Key references
Metagenetic analysis	• FastQC • DADA2 USEARCH • QIIME2, Mothur SILVA, Greengenes UNITE	• Quality control and filtering • Sequence denoising and clustering into OTUs or ASVs • Comprehensive platforms that streamline analysis workflows and databases for taxonomic classification	FastQC (Andrews, 2010), DADA2 (Callahan et al., 2015), USEARCH (Zhou et al., 2024), QIIME2 (Bolyen et al., 2019), Mothur (Schloss et al., 2009), SILVA, Greengenes, UNITE (Santamaria et al., 2012)
Metagenomic analysis	• Trimmomatic, FASTP • MEGAHIT, SPAdes • Kraken2, (Segata et al., 2012) MetaPhlAn • KEGG	• Quality control • Sequence assembly • Taxonomic classification • Functional annotation	Trimmomatic (Bolger et al., 2014), FASTP (Chen, 2023), MEGAHIT (Li et al., 2015), SPAdes (Bankevich et al., 2012), Kraken 2 (Wood et al., 2019), MetaPhIAn (Segata et al., 2012), KEGG (Kanehisa et al., 2017)
Long-read sequencing and assembly	• Flye, Canu • Medaka, Racon	• Long-read assembly • Sequence polishing	Flye (Kolmogorov et al., 2019), Canu (Koren et al., 2017), Medaka, Racon (Lee et al., 2021)
Metagenomic binning and MAGs analysis	• MetaBAT2, CONCOCT, MaxBin • CheckM, GUNC • Prokka, EggNOG-mapper	• Binning of contigs into genome bins • Quality assessment of MAGs • Genome annotation	MetaBAT2 (Kang et al., 2019), CONCOT (Alneberg et al., 2014), Maxbin (Wu et al., 2014), CheckM (Parks et al., 2015), GUNC (Orakov et al., 2021), Prokka (Seemann, 2014), EggNOG-mapper (Huerta-Cepas et al., 2017)
Functional profiling	• HUMAnN3, MEGAN, FAPROTAX, PICRUSt2	• Prediction of metabolic functions	HUMAnN3 (Beghini et al., 2021), MEGAN (Beier et al., 2017), FAPROTAX (Louca et al., 2016), PICRUSt2 (Douglas et al., 2020)
Metatranscriptomic analysis	• FASTQC, Trimmomatic, SortMeRNA • HISAT2, STAR, MEGAHIT, Trinity • Diamond, Kaiju • DESeq2, edgeR, and KEGG Mapper	• Preprocessing • Alignment and assembly • Functional annotation • Differential expression and pathway analysis	SortMeRNA (Kopylova et al., 2012), HISAT2 (Wen, 2017), STAR (Dobin et al., 2013), Trinity (Haas et al., 2013), Diamond (Buchfink et al., 2015), Kaiju (Menzel et al., 2016), DESeq2 (Anders and Huber, 2010), edgeR (Robinson et al., 2010), KEGG Mapper (Kanehisa and Sato, 2020)
WGS and AMR analysis	• Unicycler • Bakta, ROARY • CARD, ResFinder, AMRFinderPlus, ARIBA	• Genome assembly and annotation • Comparative genomics • AMR gene identification	Unicycler (Wick et al., 2017), Bakta (Schwengers et al., 2021), ROARY (Page et al., 2015), CARD (Alcock et al., 2023), Resfinder (Florensa et al., 2022), AMRFinderPlus (Feldgarden et al., 2021), ARIBA (Hunt et al., 2017)

## 4 Application of NGS in food science

### 4.1 NGS for food safety

The application of NGS in food safety assessment has significantly improved pathogen detection, microbial community analysis, and AMR surveillance (Pennone et al., 2022).

Metagenetics, primarily based on 16S rRNA gene sequencing, is used to study microbial communities in food and their processing environments. For example, Anagnostopoulos et al. (2022) and Anagnostopoulos et al. (2023) used 16S metabarcoding to analyze microbial succession in ice-stored seabream, revealing shifts in bacteria like *Pseudomonas* and *Shewanella* associated with spoilage. Similarly, Syromyatnikov et al. (2020) applied 16S sequencing to butter microbiota, detecting opportunistic pathogens missed by traditional methods. Díaz Cárdenas et al. (2024) studied street-vended foods in Ecuador, identifying dominant genera like *Acinetobacter*, *Lactococcus*, and *Vibrio*. They found twenty-nine spoilage bacteria and twenty-four opportunistic pathogens, underscoring the food safety risks in these environments and highlighting the importance of metagenetics for food safety monitoring.

WGS offers unmatched precision for examining individual microbial isolates, allowing for meticulous strain tracking during outbreaks. Bai et al. (2024) applied WGS to fresh-cut fruits and vegetables, detecting *Salmonella*, *Listeria monocytogenes*, and *E. coli* along with virulence and resistance genes. Similarly, Habib et al. (2024) found multidrug-resistant *Salmonella enterica* in chilled broiler chicken. These bacterial pathogens are among the most relevant to public health in the various food production sectors in the EU/EEA (Koutsoumanis et al., 2024). Moreover, the emergence of long-read sequencing technologies like Oxford Nanopore suggests a pathway toward real-time, portable, on-site food safety testing (Lee et al., 2024). To provide a clearer overview of key foodborne pathogens, their typical food sources, and their detection using NGS, Table 4 summarizes selected examples aligned with recent research.

**TABLE 4:** Overview of foodborne pathogens, food sources, and their detection via NGS.

Foodborne pathogen	Food source	NGS focus	Sequencing platform	Application	References
*Bacillus cereus*, *L*. *monocytogenes*, *Salmonella spp*., *Shigella spp*., *Vibrio cholerae*, *Vibrio parahaemolyticus* and *Vibrio vulnificus*	Fresh seafood	Metagenetics	Illumina	Explored the microbial diversity in seafood factories, revealing significant insights into potential pathogenic threats	Kuan et al. (2025)
*Campylobacter jejuni*	Contaminated poultry products	Transcriptomics	Illumina	Investigated the mechanism of antimicrobial formulations against *Campylobacter*	Tang et al. (2021)
*C*. *jejuni*	Retail chicken	Comparative genomics	Illumina, Nanopore	Compared genomic data between *C. jejuni* isolates to understand virulence factors and resistance mechanisms	Neal-McKinney et al. (2021)
*Cronobacter sakazakii*	Garlic extract	Transcriptomics	Illumina	Analyzed responses to antimicrobial compounds from garlic, establishing gene expression profiles	Feng et al. (2014)
*E*. *coli*	Canola sprouts	Transcriptomics	Illumina	Identified genes essential for survival in plant tissues, emphasizing iron acquisition mechanisms	Na et al. (2018)
*E. coli* HEHA16, *L. monocytogenes*, *Salmonella enterica Typhi, Cronobacter sakazakii*	Meat and meat analogues	Whole Genome Sequencing	Illumina	Investigated the dynamics of foodborne pathogens in meats using traditional microbiology and NGS for better pathogen detection	Bonaldo et al. (2023)
*L. monocytogenes*	Food grade stainless steel	Transcriptomics	Illumina	Evaluated transcriptional changes upon desiccation stress, revealing adaptation mechanisms	Kragh and Truelstrup Hansen (2020)
*L. monocytogenes*	Food processing environments	Metagenetics	Illumina	Identified diverse *Listeria spp*. in food processing facilities, linking environmental microbiota to pathogen presence	Tan et al. (2019)
Shiga toxin-producing *E. coli*	Raw palm sap Samples	Metabarcoding	Nanopore	Used nanopore sequencing to identify pathogenic species in palm sap, emphasizing NGS approaches for foodborne pathogen monitoring	Ábrahám et al. (2024)
*Vibrio spp*. and oportunistic pathogens	Street-vended foods	Metabarcoding	Illumina	Characterized the microbial composition of street vended foods	Díaz Cárdenas et al. (2024)

Beyond outbreak detection, NGS also strengthens food safety through its role in preventive quality control and source attribution. By characterizing microbial contaminants at high resolution, NGS can help detect the origin of contamination, differentiating whether it arose from raw ingredients, surfaces, or environmental niches. This technology is especially useful when multiple sources of contamination are involved, as genomic comparison of isolates from diverse sampling points can uncover distinct strain lineages or reveal shared transmission routes (Rantsiou et al., 2018; Koutsoumanis et al., 2024).

Metatranscriptomics, while less frequently deployed, introduces a unique analytical layer by targeting gene expression (RNA) rather than solely genetic potential (DNA). This approach is particularly useful for assessing spoilage mechanisms and microbial metabolism in perishable foods. For example, de Lira et al. (2020) integrated metatranscriptomics to examine histamine production in fish, which highlights its utility in connecting specific microbial functions to observable food quality deterioration. Its application in verifying the activity of foodborne viruses like norovirus Yang et al. (2024), also demonstrates its value extends beyond bacteria. This technique, therefore, provides critical insights into which microbes are metabolically active and the functions they are executing within the food matrix at a specific moment.

The widespread adoption of NGS across varied food industries from dairy (Ma et al., 2018; Yang et al., 2020; Liang et al., 2022) and meat/poultry products (Barcenilla et al., 2024; Martínez-Álvarez et al., 2025; Wang et al., 2024) to fresh produce (Solcova et al., 2021; Gu et al., 2024) attests to its flexibility, but also brings common challenges into focus. A consistent theme emerging from these diverse studies is the significant influence of processing environments on microbial ecology and the spread of AMR genes (Gaire et al., 2024; Koutsoumanis et al., 2024), alongside the risks posed by pathogens and resistance elements in raw ingredients. This implies that microbial risks are ubiquitous yet shaped by the specifics of each food type and its journey through the production chain.

For effective management, NGS monitoring strategies should be customized for different sectors, concentrating on critical control points identified via thorough environmental and product analyses. Promoting the sharing of data and insights from these varied applications, following the example of successful collaborative projects (Allard et al., 2016) will be instrumental in refining best practices and informing risk management universally.

Comparing NGS with conventional methods starkly illustrates its advantages in speed, resolution, and culture-independent analysis, facilitating more potent outbreak responses and surveillance (Forbes et al., 2017; Hilt and Ferrieri, 2022; Panwar et al., 2023). The practical impact is evident in real-world scenarios, such as the FDA resolving complex outbreaks using WGS data in regulatory decisions (Ottesen and Ramachandran, 2019; Mak et al., 2025). Nevertheless, significant hurdles impede its routine implementation: costs, the demand for specialized bioinformatics skills and absence of standardization present considerable barriers. Future progress hinges on collaborative initiatives aimed at standardizing protocols and bioinformatics workflows, substantial investment in training and infrastructure, and the creation of unambiguous guidelines for validation and data interpretation.

### 4.2 NGS for food fermentation

**FIGURE 2:**
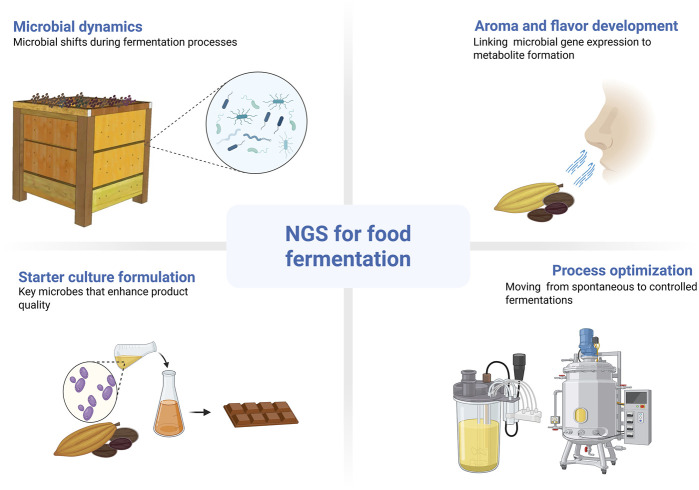
Overview NGS applications in food fermentation, including microbial community analysis, starter culture formulation, flavor development, and process optimization. Created using BioRender: https://BioRender.com.

Food fermentation enhances food products through complex microbial activities, traditionally studied with methods that were limited in scope. NGS offers a transformative approach, providing extensive insights into microbial community structure, function, and dynamics (Pothakos et al., 2020; Ferrara et al., 2021; Kim et al., 2021; Lima et al., 2022; Tamang et al., 2022; Xiong et al., 2024). The application of NGS reveals the previously hidden microbial diversity, including unculturable organisms, vital for fully understanding fermentation processes. Figure 2 summarizes the main contributions of NGS to food fermentation, including insights into microbial dynamics, starter culture development, process optimization, and the linkage between gene expression and flavor compound formation.

NGS methods like amplicon sequencing and shotgun metagenomics provide detailed taxonomic profiles (Ferrara et al., 2021; Tigrero-Vaca et al., 2022; Vaccalluzzo et al., 2022), and metatranscriptomics identifies active genes and microbes during fermentation stages (Pothakos et al., 2020; Kim et al., 2021; Xiong et al., 2024). Studies exemplify this power: identifying biogenic amine producers in soy sauce highlights NGS’s role in assessing safety aspects alongside flavor development (Kim et al., 2021); revealing microbial shifts influencing volatile compounds in olives demonstrates its utility in targeted quality improvement (Vaccalluzzo et al., 2022); and uncovering complex networks in focaccia contrasts sharply with the limited view from plating methods (Ferrara et al., 2021).

The consistent finding across fermentation studies is that NGS offers a far more comprehensive view than traditional methods, which is crucial for informed development of starter cultures. A potential gap suggested by the focus on dominant bacteria (like lactic acid bacteria) is the need for deeper investigation into the roles of sub-dominant bacteria, yeasts, and potentially other microbes; employing deeper sequencing or targeted enrichment could address this.

Functionally, metagenomics predicts the metabolic potential (Kharnaior and Tamang, 2022; Lima et al., 2022; Tamang et al., 2022), while metatranscriptomics confirms active pathways (Kim et al., 2021; Xiong et al., 2024). Linking specific microbial gene expression to metabolite formation provides direct targets for intervention (Kim et al., 2021; Kharnaior and Tamang, 2022; Xiao et al., 2022; Xiong et al., 2024). Uncovering enzymatic functions in cacao or amino acid profiles in cheonggukjang directly informs strategies for enhancing specific sensory attributes (Lima et al., 2022; Tamang et al., 2022; Tigrero-Vaca et al., 2022). A significant implication is the ability to move from spontaneous to controlled fermentations by understanding and manipulating these functional links.

Ultimately, the application of NGS profoundly shapes the future of food fermentation. Identifying key microbes and metabolic pathways facilitates the rational design and selection of starter cultures tailored for specific outcomes, such as desired flavor profiles in cacao or controlled activity in coffee (Pregolini et al., 2021; Lima et al., 2022; Tigrero-Vaca et al., 2022; Constante Catuto et al., 2024). Understanding microbial succession and function, as shown in paocai and suancai, is critical for process optimization (Xiao et al., 2022; Xiong et al., 2024). A clear direction arising from these studies is the synergy gained from multi-omics approaches; combining genomics, transcriptomics, and metabolomics offers the most holistic view (Kharnaior and Tamang, 2022; Xiong et al., 2024) and represents a key strategy to address remaining knowledge gaps about complex microbial interactions and their precise impact on final product characteristics.

### 4.3 Other NGS applications in food science

In addition to food safety and fermentation, NGS is driving innovative approaches in food authentication, traceability, and product integrity. Techniques like metagenomics and metabarcoding are being employed to ensure the quality and authenticity of food products.

Metabarcoding, is a molecular technique that enables the identification of multiple species within a mixed sample, such as bulk or environmental DNA, through high-throughput sequencing of a targeted genetic marker (Liu et al., 2020). In food science, metabarcoding plays a crucial role in species authentication, detection of adulteration, and monitoring of microbial communities (Mottola et al., 2024). For example, Detcharoen et al. (2024) used Nanopore sequencing to authenticate fish species in surimi-based products, offering a rapid and accurate method for detecting species substitution and ensuring food traceability. Additionally, Giusti et al. (2024) employed metabarcoding to uncover species mislabeling in insect-based products marketed in the EU, underscoring its value in ensuring transparency and regulatory compliance in novel food markets.

Nanopore sequencing has also proven valuable in real-time, on-site detection of contaminants in brewing processes. Kurniawan et al. (2021) developed a rapid nanopore-based platform to identify beer-spoiling bacteria directly in breweries, improving quality control and minimizing spoilage risks. In another brewing application, (Shinohara et al., 2021), utilized nanopore sequencing to identify yeast species quickly and accurately in breweries, enhancing yeast management and beer production.

Beyond these applications, NGS has also emerged as a powerful tool in studying food microbiome interactions. For example, Lerma-Aguilera et al. (2024) examined how cooking methods influence microbial diversity, while Küçükgöz et al. (2025) examined the impact of fermented beetroot ketchup on gut microbiota. Similarly, Gong et al. (2024) investigated the effects of processed oats and pinto beans on gut microbiota, demonstrating that dietary fibers from these foods promote beneficial bacteria. Additionally, Milani et al. (2025) demonstrated how bacteria present in cheese can modulate the gut microbiome, emphasizing the role of food as a vehicle for beneficial microbes. These findings underscore the growing importance of NGS in assessing the functional effects of food on human health.

### 4.4 Integration of artificial intelligence (AI) and machine learning (ML) with NGS in food science

The convergence of NGS with AI and ML is rapidly reshaping food science, offering predictive power and real-time decision-making in areas such as food safety, traceability, and microbial risk assessment (Liao et al., 2024). These approaches go beyond traditional detection and profiling, enabling the extraction of meaningful patterns from complex, high-dimensional sequencing data.

Supervised learning models, such as random forests and decision trees, have been used to predict foodborne pathogen contamination. For example, Bolinger et al. (2021) applied random forest classifiers to 16S rRNA microbiome profiles from poultry rinsates to estimate *Salmonella* contamination risk at slaughter. Their model identified specific microbial signatures as reliable proxies for pathogen presence, demonstrating the predictive value of sequencing data integrated with ML algorithms.

Explainable AI approaches are also emerging. Ince et al. (2025) combined WGS with a decision-tree model, using SHAP (SHapley Additive exPlanations) values to quantify the influence of microbiota features and temporal variables on the growth of *Clostridium perfringens* in pork. This interpretable framework offered microbiologically relevant insights, advancing food spoilage prediction and process control.

Baker et al. (2023) further demonstrated the use of ML in food safety surveillance by combining shotgun metagenomics with unsupervised clustering and supervised models to examine microbial communities and AMR genes across poultry farms and abattoirs in China. The study revealed consistent resistome profiles across sites, suggesting common selective pressures and potential routes of cross-contamination. This illustrates how AI-enhanced NGS analyses can support comprehensive surveillance of AMR and microbial hazards across the food production chain.

Beyond safety, ML tools have also been applied to food authentication. Sabater et al. (2024) combined metagenomic sequencing with supervised machine learning to trace the geographic origin of Spanish Protected Designation of Origin (PDO) honey, identifying microbial community signatures as indicators of authenticity. This approach underscores the potential of ML-integrated NGS data to support fraud detection and ensure product traceability. Similar strategies could be extended to other high-value or regulated food products, making AI-enhanced NGS an asset in modern food authentication frameworks.

In the domain of food fermentation, ML and multi-omics approaches are proving especially powerful (Li et al., 2025). integrated metagenomics sequencing and metabolomics with machine learning algorithm (logistic regression, K-nearest neighbors (KNN), and random forest) to classify abnormal stacking fermentations in sauce-flavor Baijiu. Their models successfully distinguished between types of fermentation failure, and SHAP analysis was used to identify key microbial and metabolite biomarkers driving the predictions. This study exemplifies how AI enhanced multi-omics can uncover mechanistic insights into microbial dynamics and functional disruptions, supporting both quality control and the optimization of traditional fermentation processes.

**FIGURE 3:**
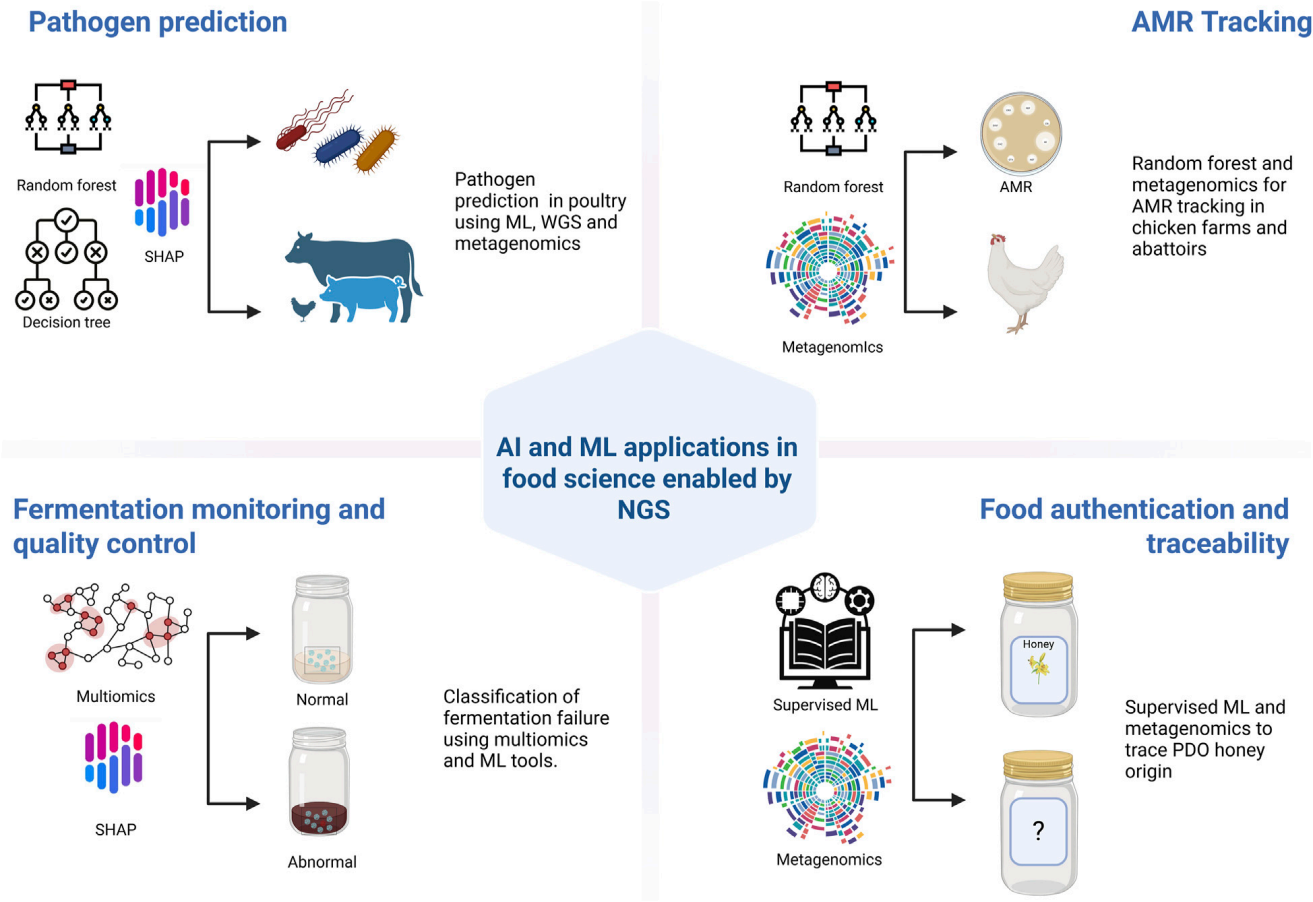
Schematic representation of AI and ML applications enabled by NGS in food science, including pathogen prediction, AMR tracking, fermentation quality control, and food authentication using models like random forest and multi-omics integration. Created using BioRender: https://BioRender.com.

Figure 3 presents a schematic representation of AI and ML applications in food science enabled by NGS, including pathogen prediction, AMR tracking, fermentation monitoring, and food authentication. The diagram highlights the integration of models such as random forest, SHAP, and supervised learning with omics data to support predictive and diagnostic capabilities across food systems.

Despite these advances, challenges remain. Many models are dataset-dependent, limiting generalizability across food systems. The need for standardized pipelines, validated microbial markers, and robust training datasets is critical. Furthermore, regulatory acceptance and interpretability of AI outputs are ongoing concerns.

Looking forward, integrating multi-omics data (e.g., genomics, transcriptomics, metabolomics) with hybrid AI models may provide a more holistic understanding of microbial behavior, food spoilage mechanisms, and safety risks. As sequencing technologies and computational methods evolve, AI and ML will become increasingly central to precision food safety, real-time diagnostics, and intelligent quality assurance.

## 5 Conclusion and future research

NGS has transformed food science by enabling culture-independent pathogen detection, AMR surveillance, and microbial community profiling, significantly improving food safety and outbreak prevention. Compared to traditional methods, NGS offers greater sensitivity and a broader scope for detecting pathogens, spoilage organisms, and resistance genes across various food matrices.

In food fermentation, NGS has deepened our understanding of microbial interactions, metabolic pathways, and flavor formation, optimizing starter cultures and enhancing product quality. Similarly, food authentication and traceability have benefited from metabarcoding and metagenomics, improving fraud detection and product integrity.

Despite these advantages, challenges such as high sequencing costs, complex data interpretation, and the need for standardized bioinformatics workflows hinder widespread adoption. Addressing these requires harmonized analytical pipelines, improved computational tools, detailed and clear regulatory guidelines. Integrating multi-omics approaches and leveraging AI will further enhance microbial analysis, food authenticity verification, and risk assessment.

Future research should focus on advancing real-time, on-site sequencing technologies for rapid pathogen detection in food processing environments. Portable platforms like nanopore sequencing could revolutionize contamination monitoring and outbreak response. Additionally, microbiome-based food safety strategies and predictive modeling using microbial signatures may help prevent contamination and assess food quality. Expanding NGS applications to emerging food technologies, including alternative proteins and novel fermented foods, will be critical for ensuring their safety and quality. Establishing globally accepted regulatory frameworks will further facilitate industry adoption and standardization.

As NGS technology evolves, interdisciplinary collaboration will be key to maximizing its impact on food safety, quality assurance, and innovation, contributing to a safer and more sustainable food system.
